# Hospitals in India

**Published:** 1894-01-06

**Authors:** 


### HOSPITALS IN INDIA.

### I.—THE BENGAL PRESIDENCY.

(7.) DISPENSARIES AND CHARITABLE INSTITUTIONS OF THE NORTH-WESTERN PROVINCES AND OUDH.

At the end of 1891 there were 299 hospitals and dispensaries of all classes in operation in these provinces. Of these institutions two were Class I., or State hospitals; 241 were Class II., or District Board dispensaries; 33 were Class III. A, or private dispensaries; and 23 belonged to Class III. B, or State-aided dispensaries. The number of beds available was 2,900, and of these 2,253·44 were occupied on an average daily, the total number of indoor patients being 54,372. Of this number 33,171 were cured, 9,679 were elieved, 6,075 were discharged otherwise, and 3,357 died, the ratio of deaths per cent. of total treated being 6·17, as compared with 5 87 in the previous year. The increase in the death-rate is attributed to the more serious nature of the cases admitted into the wards, and also to a vast increase in the number of important surgical operations performed, which rose from 19,420 in 1890 to 23,321 in 1891. Minor surgical operations also rose from 123,366 to 131,642. The results of the major operations appear to be highly satisfactory, 17,745 cures having been effected, while 4,315 were relieved and discharged, and only 330 died; the remaining 931 were still under treatment on the last day of the year. The number of out-door patients treated was 3,012,662, of whom 2,808,250 attended personally, and 204,412 were represented by friends The daily average attendance of this class was 18,394.04, as compared with 17,875 11 in the previous year. In-door and out-door patients combined thus numbered 3,067,034 in 1891, as compared with 2,427,733 in 1890, an increase of 26 3 per cent.

It is satisfactory to note that the number of female patients

**increased by more than 37 per cent. Excluding the Maternity Hospital at Agra, there were twenty separate hospitals and dispensaries for the exclusive use of women, borne on the list in 1891, and these provided accommodation for 256 in-door patients as compared with an average daily number including children, of 213·87. The provision of additional hospitals for women, and the training of a female staff to assist in the female hospitals, is now being considered by Government, the twenty institutions referred to above belonging mainly to the Dufferin Fund Association. The principal points in the scheme under consideration are (1) accommodation for females, whether as in or out patients, in buildings (other than those in which male patients are received, and entirely separate from them) which should provide a sufficient number of wards for in patients, together with private and seperate accommodation for three or four inmates ; (2) competent female hospital assistants ; and (3) qualified nurses and midwives for practice in the hospital and in the adjoining city.**

**Appended is the briefest possible summary of the financial results of the two years 1890 and 1891 ; the percentage of the total cost paid by government was 48·34, and the average cost of each diet was 1a. 1¾p.**

**Table Showing the Financial Returns for the Hospitals and Dispensaries in the N.W. Provinces and Oudh.**

| | 1890. Rs. | A. | P. | | 1891. Rs. | A. | P. |
|---|---|---|---|---|---|---|---|
| Cash balance on 1st Jan. | 132,673 | 8 | 3 | ... | 146,847 | 14 | 10 |
| Income from all sources | 479,563 | 11 | 1 | ... | 544,066 | 9 | 11 |
| Total ... ... | 612,237 | 3 | 4 | ... | 690 914 | 8 | 9 |
| Expenditure ... ... | 465,025 | 9 | 0 | ... | 543,695 | 15 | 0 |
| Cash balance on 31st Dec. | 147,211 | 10 | 4 | ... | 147,218 | 9 | 9 |

**The difference between the cash balance on the 1st of January, 1891, and that shown to exist on the 31st of December, 1890, is owing to subsequent corrections in the accounts of the year ending on the latter date.**

